# Branched-chain amino acid supplementation restores reduced insulinotropic activity of a low-protein diet through the vagus nerve in rats

**DOI:** 10.1186/s12986-017-0215-1

**Published:** 2017-09-15

**Authors:** Mami Horiuchi, Tomoya Takeda, Hiroyuki Takanashi, Yori Ozaki-Masuzawa, Yusuke Taguchi, Yuka Toyoshima, Lila Otani, Hisanori Kato, Meri Sone-Yonezawa, Fumihiko Hakuno, Shin-Ichiro Takahashi, Asako Takenaka

**Affiliations:** 1 Department of Agricultural Chemistry, School of Agriculture, Meiji University, Kanagawa, Japan; 2 Institute for Advanced Medical Sciences, Nippon Medical School, Kanagawa, Japan; 3 Corporate Sponsored Research Program “Food for Life” Organization for Interdisciplinary Research Projects, The University of Tokyo, Tokyo, Japan; 4 Department of Animal Sciences and Applied Biological Chemistry, Graduate School of Agriculture and Life Sciences, The University of Tokyo, Tokyo, Japan; 5 Department of Chemistry and Life Science, College of Bioresource Sciences, Nihon University, Kanagawa, Japan

**Keywords:** Branched-chain amino acid, Low-protein diet, Insulin, Vagotomy, Rat

## Abstract

**Background:**

Previously, we reported that a low-protein diet significantly reduced insulin secretion in response to feeding within 1 h in rats, suggesting that the insulinotropic effect of dietary protein plays an important role in maintaining normal insulin release. The current study aimed to elucidate whether deficiency of certain amino acids could diminish the insulinotropic activity and to investigate whether reduced insulin secretion in response to a low-protein diet is restored by supplementation with certain amino acids.

**Methods:**

First, we fed male Wistar rats (5–6 rats per group) with diets deficient in every single amino acid or three branched-chain amino acids (BCAAs); within 1–2 h after the onset of feeding, we measured the plasma insulin levels by using an enzyme-linked immunosorbent assay (ELISA). As insulin secretion was reduced in BCAA-deficient groups, we fed low-protein diets supplemented with BCAAs to assess whether the reduced insulin secretion was restored. In addition, we treated the pancreatic beta cell line MIN6 with BCAAs to investigate the direct insulinotropic activity on beta cells. Lastly, we investigated the effect of the three BCAAs on sham-operated or vagotomized rats to assess involvement of the vagus nerve in restoration of the insulinotropic activity.

**Results:**

Feeding a low-protein diet reduced essential amino acid concentrations in the plasma during an absorptive state, suggesting that reduced plasma amino acid levels can be an initial signal of protein deficiency. In normal rats, insulin secretion was reduced when leucine, valine, or three BCAAs were deficient. Insulin secretion was restored to normal levels by supplementation of the low-protein diet with three BCAAs, but not by supplementation with any single BCAA. In MIN6 cells, each BCAA alone stimulated insulin secretion but the three BCAAs did not show a synergistic stimulatory effect. The three BCAAs showed a synergistic stimulatory effect in sham-operated rats but failed to stimulate insulin secretion in vagotomized rats.

**Conclusions:**

Leucine and valine play a role in maintaining normal insulin release by directly stimulating beta cells, and supplementation with the three BCAAs is sufficient to compensate for the reduced insulinotropic activity of the low-protein diet, through the vagus nerve.

## Background

It has been reported that dietary protein deficiency induces impaired insulin secretion in response to glucose or feeding. Glucose-induced insulin secretion was reduced in protein-calorie malnourished patients [1] and rats fed a low-protein diet for more than 1 week [2–5]. Insulin secretion was also impaired in isolated pancreatic islets from protein-restricted rats [6], and decreased islet size and loss of islet beta cells [7, 8] have been reported as mechanisms for the impaired insulin secretion. These results demonstrate that intake of a protein deficient diet over a period of weeks reduces insulin secretion through decreased size of the islet and impaired function of the beta cells.

On the other hand, we have reported that a low-protein diet significantly reduced insulin secretion in response to feeding within 1 h [9], which is probably caused by the low insulinotropic activity of the diet itself. This means that insulinotropic effects of dietary protein also play important roles in maintaining normal insulin release during and soon after meals. Insulinotropic activity of dietary protein on glucose-induced insulin secretion in humans has already been reported in the 1960s [10, 11]. In addition to the effect of oral ingestion of protein, Floyd et al. demonstrated that intravenous co-administration of an amino acid mixture with glucose stimulated insulin secretion [12], which means amino acids in plasma have important roles to stimulate insulin secretion. They also demonstrated that the combination of specific amino acids strongly increased plasma insulin [13]. Later on, it was demonstrated in humans that co-ingestion of leucine (Leu) and phenylalanine (Phe) with wheat protein hydrolysate and carbohydrate strongly increased plasma insulin [14]. These results suggest that insulin secretion in response to a meal is enhanced by specific amino acids in plasma and that lack of the insulinotropic effect of amino acids could be the cause for the reduced insulin secretion in response to a low-protein diet.

Insulin secretion from pancreatic beta cells is basically stimulated by glucose; when ATP is produced through glucose oxidation, ATP-sensitive K^+^ channels are opened, causing membrane depolarization of the beta cells. This depolarization induces translocation of insulin secretory vesicles and successive insulin secretion from the cells by exocytosis [15]. In addition to this mechanism, insulin secretion is stimulated by gastric peptides such as glucagon-like peptide (GLP)-1, which are secreted from the intestine in response to glucose or nutrients, and stimulates insulin secretion from pancreatic beta cells via specific receptors [16]. Furthermore, the vagus nerve also regulates insulin secretion from pancreatic beta cells via the M3 muscarinic receptor [17, 18].

The effects of amino acids on insulin secretion have been investigated using pancreatic beta cells. Positively charged amino acids such as arginine (Arg) and lysine (Lys) are known to stimulate insulin secretion because these amino acids induce membrane depolarization when transported into the cells [19, 20]. Alanine (Ala) and proline (Pro) are co-transported with Na^+^, induce membrane depolarization, and augment glucose-dependent insulin secretion [21, 22]. Some amino acids such as Ala, Leu, Pro, and glutamine (Gln) serve as fuels for ATP production via tricarboxylic acid (TCA) cycle and stimulate insulin secretion [23, 21]. Among these amino acids, Leu is considered to be the strongest stimulator of insulin secretion as it activates glutamate dehydrogenase, the enzyme that catalyzes glutamate to α-ketoglutarate and leads ATP production [24]. Moreover, Leu is a potent stimulator of mammalian target of rapamycin (mTOR) [25, 26] and is reported to stimulate insulin secretion through activation of mTOR signaling [27].

Here, we aimed to clarify whether there are specific amino acids whose deficiency causes the impaired insulinotropic activity of a low-protein diet and whether the regulation involves the vagus nerve. First, we fed rats with a single amino acid-deficient diet and elucidated which amino acid deficiency reduced insulin secretion. We then studied whether supplementation of a low-protein diet with any amino acid could restore the impaired insulin secretion. Lastly, we investigated the insulinotropic mechanisms of the amino acids. The direct stimulation of insulin secretion from cultured beta cells and the involvement of the vagus nerve in amino acid stimulation of insulin secretion in vivo were examined.

## Methods

### Materials

Casein, α-cornstarch, cellulose, vitamin mixture and mineral mixture were purchased from Oriental Yeast Co., Ltd. (Tokyo, Japan). MIN6 cells [28] were provided by Dr. J Miyazaki, Graduate School of Medicine, Osaka University (MTA13–148).

### Animals

Male Wistar rats of 6 or 7 weeks old (160–210 g) were purchased from Japan Laboratory Animals Inc. (Tokyo, Japan) and housed in a room under a 12 h light/dark cycle (06:00–18:00) at a temperature of 22–24 °C. Rats were allowed free access to standard chow (MF; Oriental Yeast Co., Ltd.) and water for 2 or 3 days to acclimate to the housing condition. Before animal experiments, the rats were fed a control diet containing 20% casein as a protein source (20P) from 8:00 to 16:00 for 4 days and trained for the time-restricted feeding that was applied to the all animal experiments. The composition of the 20P diet is shown in Table 1.

**Table 1:** Composition of the amino acid supplemented diets

			Supplemented amino acids			
	20P	5P	Val	Leu	Ile	BCAA
α-Cornstarch	43.7	51.6	51.6	51.6	51.6	51.6
Sucrose	21.8	25.8	25.8	25.8	25.8	25.8
Cellulose	5	5	5	5	5	5
AIN93 vitamin mixture	1	1	1	1	1	1
AIN93G mineral mixture	3.5	3.5	3.5	3.5	3.5	3.5
Corn oil	5	5	5	5	5	5
*Protein and amino acids*						
Casein	20	5	5	5	5	5
L-Valine			0.96			0.96
L(+)-Isoleucine					0.86	0.86
L-Leucine				1.31		1.31
L-Glutamic acid		3.14	2.18	1.82	2.27	
*Total amount of protein and amino acids*	20	8.1	8.1	8.1	8.1	8.1

(g/100 g diet)

20P control diet containing 20% casein, 5P low-protein diet containing 5% casein Val valine, Leu leucine, Ile isoleucine, BCAA branched-chain amino acid (Val + Leu + Ile) AIN, American Institute of Nutrition

### Animal experiments

#### Experiment 1: Effect of feeding low-protein diet on plasma amino acid concentrations

After overnight fast for 16 h, the rats were fed 20P or a low-protein diet containing 5% casein (5P) for 30 min. Blood samples from artery and portal vein were drawn into a syringe containing heparin under inhalational anesthesia with isoflurane for measurement of plasma free amino acid concentrations. The composition of the 5P diet is shown in Table 1.

#### Experiment 2: Effect of single amino acid deprivation on insulin secretion

After overnight fast for 16 h, the rats were fed a control diet containing 20% amino acid mixture simulating casein (20AA), or diets deprived of single amino acid. The composition of the 20AA is shown in Table 2. Single amino acid-deprived diets were prepared by removing each amino acid from 20AA and supplementing with the same amount of glutamic acid (Glu), which is the most abundant nonessential amino acid in 20AA, to make amino acid contents of the all experimental diets same. Blood samples were obtained by tail incision and drawing into heparinized tubes at 0, 1, and 2 h after feeding to measure plasma insulin and glucose levels. Plasma was separated by centrifugation at 700×*g* for 5 min at 4 °C and frozen at −80 °C until use.

**Table 2:** Composition of the amino acid-restricted diets

			Restricted amino acid			
	20AA	5AA	Val	Leu	Ile	BCAA
α-Cornstarch	43.7	53.7	43.7	43.7	43.7	43.7
Sucrose	21.8	26.8	21.8	21.8	21.8	21.8
Cellulose	5	5	5	5	5	5
AIN93 vitamin mixture	1	1	1	1	1	1
AIN93G mineral mixture	3.5	3.5	3.5	3.5	3.5	3.5
Corn oil	5	5	5	5	5	5
*Amino Acids*						
L-Aspartic acid	1.13	0.28	1.13	1.13	1.13	1.13
L(−)-Threonine	0.78	0.20	0.78	0.78	0.78	0.78
L-Serine	1.09	0.27	1.09	1.09	1.09	1.09
L-Glutamic acid	4.1	1.03	5.06	5.51	4.96	7.24
L(−)-Proline	2.14	0.54	2.14	2.14	2.14	2.14
Glycine	0.36	0.09	0.36	0.36	0.36	0.36
L-Alanine	0.54	0.14	0.54	0.54	0.54	0.54
L-Valine	1.28	0.32	0.32	1.28	1.28	0.32
L(−)-Cystine	0.07	0.02	0.07	0.07	0.07	0.07
L-Methionine	0.57	0.14	0.57	0.57	0.57	0.57
L(+)-Isoleucine	1.15	0.29	1.15	1.15	0.29	0.29
L-Leucine	1.75	0.44	1.75	0.44	1.75	0.44
L-Tyrosine	1.09	0.27	1.09	1.09	1.09	1.09
L(−)-Phenylalnine	1.01	0.25	1.01	1.01	1.01	1.01
L(+)-Lysine monohydrochloride	1.42	0.36	1.42	1.42	1.42	1.42
L-Histidine	0.52	0.13	0.52	0.52	0.52	0.52
L-Arginine	0.73	0.18	0.73	0.73	0.73	0.73
L-Tryptophan	0.26	0.07	0.26	0.26	0.26	0.26
*Total amount of amino acids*	20	5	20	20	20	20

(g/100 g diet)

20AA control diet containing 20% amino acid mixture, 5AA low amino acid diet containing 5% amino acid mixture, Val valine, Leu leucine, Ile isoleucine, BCAA branched-chain amino acid (Val + Leu + Ile), AIN American Institute of Nutrition

#### Experiment 3: Effect of branched-chain amino acid restriction on insulin secretion

After overnight fast for 16 h, the rats were fed the 20AA diet, the low amino acid diet with 5% amino acid mixture (5AA) or diet with restricted amount of branched-chain amino acids (BCAAs) (valine (Val), Leu, isoleucine (Ile), or three BCAAs). In contrast to the amino acid-deprived diets used in Experiment 2, BCAA-restricted diets were prepared by reducing the content of BCAA to 25% of the 20AA and supplementing with the same amount of Glu, which makes total amino acid content of the BCAA-restricted diets same as 20AA. The composition of the diets is shown in Table 2. Blood samples were collected at 0, 1, and 2 h after feeding and prepared for measurement of plasma insulin and glucose concentrations as described in Experiment 1. On the next day of the blood sampling described above, blood samples from artery were drawn into a syringe containing heparin under inhalational anesthesia with isoflurane for measurement of plasma free amino acid concentrations, 30 min after onset of feeding.

#### Experiment 4: Effect of branched-chain amino acid supplementation to the low-protein diet on insulin secretion

After overnight fast for 16 h, the rats were fed on 20P diet, 5P diet, or 5P diet supplemented with Val, Leu, Ile, or three BCAAs. BCAA-supplemented diets were prepared by adding BCAA to the level of 20P, and Glu was added to make the amino acid content of 5P and BCAA-supplemented diets same. The composition of the diets is shown in Table 1. Blood samples were collected and prepared for measurement of plasma insulin, glucose, and free amino acids concentrations as described in Experiments 2 and 3.

#### Experiment 5: Effect of vagotomy on the insulinotropic effect of branched-chain amino acids

Vagotomy of 6 weeks old male Wistar rats was performed under anesthesia with sodium pentobarbital (64.8 mg/kg intraperitoneally, Somnopentyl; Kyoritsu Pharmaceutical Co. Ltd., Tokyo, Japan). The dorsal and ventral branches of the vagus nerve, which run along the esophagus, were exposed and removed (*n* = 39). Sham vagotomy was conducted by the same surgical procedure except for the removal of the vagus nerve (*n* = 14). After vagotomy, rats were fed 20P from 8:00 to 16:00 every day until the beginning of the experiment. Five days after vagotomy, cholecystokinin (CCK) test of satiety was performed to verify surgical success; after 16 h fasting, CCK (CCK-octapeptide; Peptide Institute, Inc., Osaka, Japan) was injected intraperitoneally (1.6 μg/100 g BW), and food consumption during the subsequent 10 min was measured [29]. Rats without the satiety effect of CCK were selected as vagotomized and used in the experiment (*n* = 26). Two days after the CCK test, rats were deprived of food for 16 h and fed 20P, 5P, 5P supplemented with three BCAAs, or 5P supplemented with Leu for 1 h. Blood samples were collected at 0, 0.5, and 1 h after feeding and prepared for measurement of plasma insulin and glucose concentrations as described in Experiments 2 and 3.

#### Experiment 6: Effect of reduced food consumption on insulin secretion

After overnight fast for 16 h, rats were divided into two groups and fed 2.5 g of 20P or fed 20P ad libitum for 1 h. Blood samples were obtained by tail incision at 0, 0.5, and 1 h after feeding and prepared for measurement of plasma insulin and glucose concentrations as described in Experiments 2 and 3.

### Plasma analysis

After protein was precipitated and removed from plasma by adding equal amount of 3% trichloroacetic acid, free amino acid concentrations were measured by HPLC amino acid analyzer (Hitachi High-Technologies Corporation, Tokyo, Japan). Plasma glucose and insulin concentrations were measured using Glucose CII-test kit (Wako Pure Chemical Industries, Ltd., Osaka, Japan) and rat insulin enzyme-linked immunosorbent assay (ELISA) kit (Shibayagi Co., Ltd., Gunma, Japan), respectively.

### Cell experiments

MIN6 cells were maintained in Dulbecco’s Modified Eagle’s medium (DMEM) high glucose (25 mM) supplemented with 20% heat-inactivated fetal bovine serum, 5 μL/L 2-mercaptoethanol, 100 units/mL penicillin, and 100 μg/mL streptomycin and incubated in humidified 5% CO_2_ at 37 °C. Subconfluent cells (passages 24 and 25) were preincubated in Hepes–Krebs buffer containing 3 mM glucose without or with amino acids for 1 h and after washing twice with Hepes–Krebs buffer, cells were incubated in the same medium containing 3 mM or 25 mM glucose for another 1 h. Insulin in the medium was measured using a mouse insulin ELISA kit (Shibayagi Co., Ltd.), and the concentrations were normalized to the protein content of the cell lysate determined by using Bio-Rad Protein Assay Kit (Bio-Rad, Hercules, CA, USA).

### Statistics

Results are expressed as means ± S.E.M. All datasets were checked for normality and *F*-test was performed for normally distributed data. Mann–Whitney *U*-test was used for skewed data. Homoscedastic and heteroscedastic data were evaluated by unpaired Student’s *t*-test and Unpaired Welch’s *t*-test, respectively. Two-way analysis of variance (ANOVA) was performed for the results of Experiment 5. The significance level was set at *P* < 0.05. All statistical analyses were performed using Excel Statistics 2008 (Social Survey Research Information Co., Ltd., Tokyo, Japan).

## Results

### Effect of feeding low-protein diet on plasma amino acid concentrations (experiment 1)

The amount of food consumed during 30 min of the feeding period was not different between the groups (20P, 2.98 ± 0.03 g; 5P, 3.17 ± 0.02 g). Concentrations of most essential amino acids (His, Thr, Val, Met, Ile, and Leu) and some non-essential amino acids (Ser, Ala, and Tyr) were significantly reduced in 5P compared with 20P in both portal vein and artery (Table 3).

**Table 3:** Effect of protein restriction on plasma amino acid concentrations

	Portal vein						Artery					
	20P			5P			20P			5P		
Asp	57.88	±	9.32	47.36	±	9.88	70.44	±	2.39	62.57	±	2.38
Glu	119.36	±	16.87	81.34	±	9.87	94.56	±	5.84	85.06	±	9.38
Ser	354.58	±	49.84	208.98	±	16.87*	250.04	±	21.28	182.79	±	11.1*
Asn	135.76	±	22.13	73.9	±	5.94	74.01	±	6.05	49.98	±	1.93**
Gly	282.54	±	49.93	213.72	±	15.02	169.47	±	27.66	131.28	±	12.83
Gln	3.49	±	1.41	3.38	±	1.42	9.8	±	2.77	7.38	±	0.91
His	138.23	±	12.35	90.87	±	8.07*	115.56	±	6.64	88.99	±	3.48**
Thr	89.45	±	7.13	65.81	±	4.53*	82.95	±	6.33	60.75	±	2.38*
Ala	1222.46	±	139.25	810.39	±	53.87*	711.78	±	16.09	636.44	±	18.72*
Arg	54.32	±	18.01	53.58	±	8.98	30.21	±	5.36	30.61	±	2.08
Pro	9.15	±	0.92	6.88	±	0.27	22.5	±	4.72	15.96	±	2.64
Tyr	145.58	±	12.16	86.78	±	5.74**	123.82	±	6.96	80.86	±	2.90**
Val	303.78	±	27.91	168.17	±	9.07**	242.19	±	12.58	158.55	±	5.34**
Met	78.8	±	7.18	44.08	±	1.78**	66.81	±	3.17	46.28	±	1.82**
Cys	32.53	±	4.03	30.15	±	3.76	3.27	±	0.32	2.65	±	0.4
Ile	167.87	±	18.84	81.74	±	3.19**	116.07	±	6.09	73.87	±	2.35**
Leu	235.63	±	24.88	118.03	±	6.04**	173.42	±	8.99	109.25	±	3.68**
Phe	101.12	±	9.20	66.48	±	3.13	80.37	±	2.43	65.45	±	1.72**
Trp	178.12	±	94.76	36.82	±	7.33	44.32	±	11.76	32.24	±	4.67
Lys	290.25	±	62.6	187.54	±	28.1	235.73	±	43.59	130.83	±	11.21

(nmol/mL)

*p < 0.05, **p < 0.01 vs. 20P

Amino acid abbreviations: Ala alanine, Arg arginine, Asn asparagine, Cys cystine, Gln glutamine, Glu glutamate, Gly glycine, His histidine, Ile isoleucine, Leu leucine, Lys lysine, Met methionine, Phe phenylalanine, Pro proline, Ser serine, Thr threonine, Trp tryptophan, Tyr tyrosine, Val valine, 20P control diet containing 20% casein, 5P low-protein diet containing 5% casein

### Effect of single amino acid deprivation on insulin secretion (experiment 2)

**Fig. 1:**
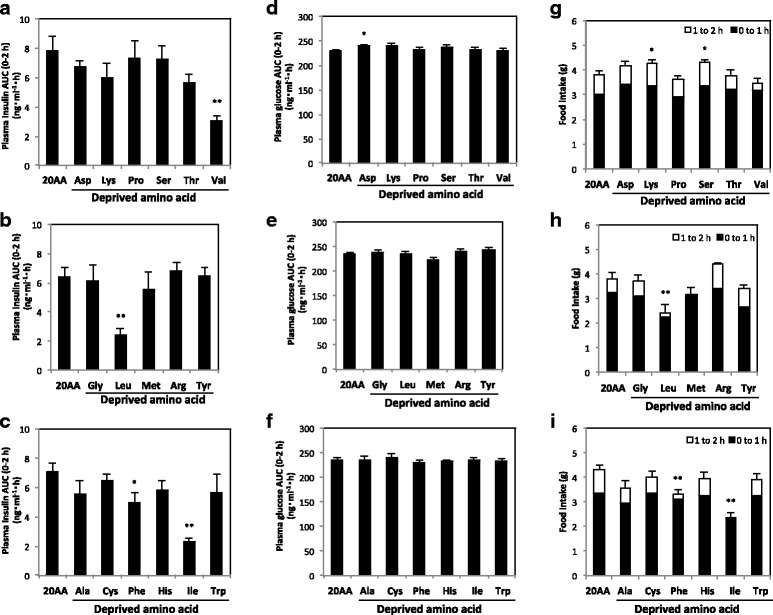
Plasma insulin and glucose concentrations and food consumption during feeding amino acid-deprived diets in rats. Three animal experiments were performed by feeding a control diet with 20% amino acids (20AA) or a diet deprived of single amino acid for 8 h; Asp, Lys, Pro, Ser, Thr, or Val (A, D, G), Gly, Leu, Met, Arg, or Tyr (B, E, H), and Ala, Cys, Phe, His, Ile, or Trp (C, F, I). Plasma insulin and glucose concentrations from 0 to 2 h after feeding are shown as AUC (**a**–**c** and **d**–**f**, respectively), and food consumptions for 0–1, 1–2 h after feeding are shown as a cumulative bar chart (**g**–**i**). Values are expressed as means ± S.E.M. (*n* = 5 in each group). Results of statistical analysis are given above the graphs (*P < 0.05; **P < 0.01 vs. 20AA). For the food consumption, cumulative value of 0–2 h after feeding was analyzed statistically

Plasma insulin after feeding single amino acid-deprived diet was measured. Insulin secretion was significantly reduced by feeding Val, Leu, Ile, and Phe-deprived diets compared with 20AA, and the reduction was remarkable (*P* < 0.01) in Val, Leu, and Ile-deprived groups (Fig. 1a–c). Plasma glucose concentration was not influenced by any single amino acid deprivation except for the increase in Asp-deprived group (Fig. 1d–f). Food intakes within 2 h of feeding were significantly lower in Leu, Phe, and Ile-deprived groups compared with the 20AA group (Fig. 1g–i).

### Effect of BCAA restriction on insulin secretion (experiment 3)

**Fig. 2:**
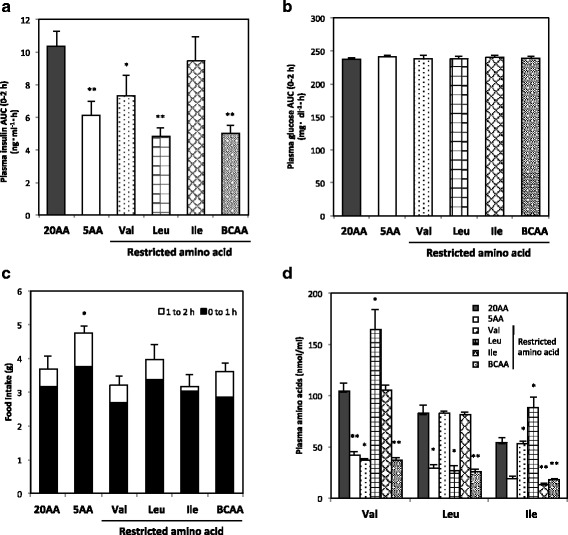
Plasma insulin, glucose, and amino acid concentrations and food consumption during feeding BCAA- restricted diets in rats. Rats were fed a control diet with 20% amino acid (20AA), a low-protein diet with 5% amino acid (5AA), or diets with reduced amount of Val, Leu, Ile, or three BCAAs (BCAA) for 8 h. Plasma insulin and glucose concentrations from 0 to 2 h after feeding are shown as AUC (**a** and **b**, respectively), food consumptions for 0–1, 1–2 h after feeding are shown as a cumulative bar chart (**c**), and plasma BCAA concentrations 30 min after feeding are also shown (**d**). Values are expressed as means ± S.E.M. (n = 5 in each group). Results of statistical analysis are given above the graphs (*P < 0.05; **P < 0.01 vs. 20AA)

Effect of Val, Leu, Ile, or three BCAA restriction to 25% of the control on insulin secretion was measured together with that of all amino acid restriction (5AA). Insulin secretion was reduced significantly (*P* < 0.01) by restriction of Leu, three BCAAs, or all amino acids without reducing plasma glucose concentration (Fig. 2a, b). In contrast to the reduced food consumption by amino acid deprivation in Experiment 1, amino acid restriction did not reduce food intakes (Fig. 2c). Arterial plasma concentrations of Val, Leu, and Ile were significantly lower in 5AA and each amino acid-restricted group, and Leu restriction increased plasma Val and Ile (Fig. 2d).

### Effect of BCAA supplementation to low-protein diet on insulin secretion (experiment 4)

**Fig. 3:**
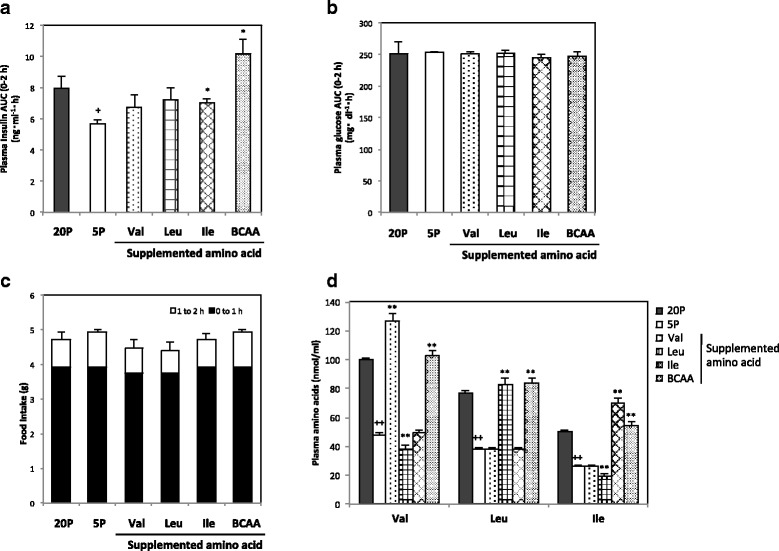
Plasma insulin, glucose, and amino acid concentrations and food consumption during feeding BCAA-supplemented diets in rats. Rats were fed a control diet with 20% casein (20P), a low-protein diet with 5% casein (5P), or diets supplemented with Val, Leu, Ile, or three BCAAs (BCAA) to 5P for 8 h. Plasma insulin and glucose concentrations from 0 to 2 h after feeding are shown as AUC (**a** and **b**, respectively), food consumptions for 0–1, 1–2 h after feeding are shown as a cumulative bar chart (**c**), and plasma BCAA concentrations 30 min after feeding are also shown (**d**). Values are expressed as means ± S.E.M. (n = 5 in each group). Results of statistical analysis are given above the graphs (^+^ *P* < 0.05, ^++^ *P* < 0.01 vs. 20P; *P < 0.05, **P < 0.01 vs. 5P)

We next examined whether impaired insulin secretion was restored by supplementation of BCAAs to a low-protein diet (5P). Although supplementation of Ile increased insulin secretion significantly, supplementation of three BCAAs synergistically increased insulin secretion and fully restored the reduced secretion of 5P compared with 20P (Fig. 3a). Plasma glucose and food intake were not affected by BCAA supplementation (Fig. 3b, c). Concentrations of Val, Leu, and Ile in arterial plasma were significantly lower in 5P than in 20P, and the concentrations of supplemented amino acids were restored to or higher than the level of 20P (Fig. 3d).

### Effect of amino acids on insulin secretion in MIN6 cells

**Fig. 4:**
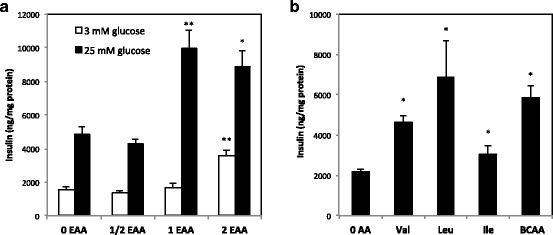
Effect of amino acids on insulin secretion from MIN6 cells. Subconfluent cells were preincubated in Hepes–Krebs buffer containing 3 mM glucose without or with amino acids for 1 h and incubated in the same medium containing 3 mM or 25 mM glucose for another 1 h. In addition to the medium without amino acids (0EAA in **a** and 0AA in **b**), amino acid-supplemented media were used; (**a**) 0AA supplemented with essential amino acids at half, equal or twice the concentration of minimum essential medium (MEM) (1/2EAA, 1EAA, or 2EAA, respectively), (**b**) 0AA supplemented with Val, Leu, Ile, or three BCAAs twice the concentration of MEM. Insulin concentration in the medium was normalized by protein content of the cell lysates. Values are expressed as means ± S.E.M. (*n* = 6 in each group). Results of the statistical analysis are given above the graphs (*P < 0.05, **P < 0.01 vs. 0EAA of the same glucose concentration or 0AA)

The direct effect of amino acid on insulin secretion from pancreatic beta cells was examined using the MIN6 cell line. High glucose at 25 mM enhanced insulin secretion regardless of essential amino acid concentrations in the medium, and high amino acid (2AA) increased insulin secretion in low-glucose medium (Fig. 4a). Deprivation or restriction of all essential amino acids (0EAA, 1/2EAA) reduced glucose-induced insulin secretion (Fig. 4a). We next examined whether BCAA directly stimulates insulin secretion in high-glucose medium and demonstrated that each of the three BCAAs stimulated insulin secretion; however, synergistic insulinotropic effect of the three BCAAs was not observed in MIN6 cells (Fig. 4b).

### Effect of vagotomy on the insulinotropic effect of BCAA (experiment 5)

**Fig. 5:**
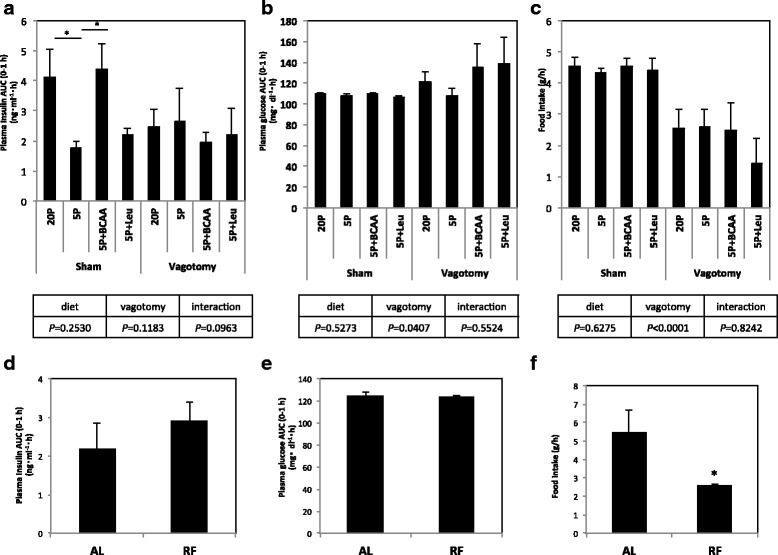
Effect of vagotomy and food restriction on plasma insulin, glucose, and amino acid concentrations in rats. **a**–**c** Vagotomized or sham-operated rats were fed a control diet with 20% casein (20P), a low-protein diet with 5% casein (5P), 5P supplemented with three BCAAs (5P + BCAA), or 5P supplemented with Leu (5P + Leu) for 1 h. Plasma insulin and glucose concentrations from 0 to 1 h after feeding are shown as AUC (**a** and **b**, respectively), and food consumption for 1 h after feeding is also shown (**c**). Values were expressed as means ± S.E.M. (sham 20P, *n* = 4; sham 5P, n = 5; sham 5P + BCAA, n = 5; sham 5P + Leu, n = 4; vagotomy 20P, *n* = 7; vagotomy 5P, *n* = 3; vagotomy 5P + BCAA, n = 4; vagotomy 5P + Leu, n = 3). Differences between 20P vs. 5P, 5P vs. 5P + BCAA, and 5P vs. 5P + Leu of the same operation group were statistically analyzed, and the results are given above the graphs (*P < 0.05). In addition, *P*-value of two-way ANOVA is shown below each graph. **d**–**f** Rats were fed a control diet with 20% amino acid (20AA) ad libitum (AL) or restricted to 2.5 g (RF) for 1 h. Plasma insulin and glucose concentrations from 0 to 1 h after feeding are shown as AUC (**d** and **e**, respectively), and food consumptions for 0–1 h after feeding are also shown (**c**). Values are expressed as means ± S.E.M. (AL, n = 3; FR, n = 4 in each group). Results of statistical analysis are given above the graphs (*P < 0.05 vs. AL)

Some rats in the vagotomized groups had higher plasma insulin than the measuring range and were excluded from the results shown in Fig. 5. Impaired insulin secretion in 5P and Leu-supplemented groups and the effect of three BCAAs supplementation to restore insulin secretion in sham-operated groups were comparable to the results shown in Experiment 4 (Fig. 5a). Insulinotropic effect of glucose in 5P was not affected by vagotomy; however, the insulinotropic effect of amino acids in 20P and 5P + BCAA was not observed in vagotomized rats (Fig. 5a). Plasma glucose was not affected by diet and was increased by vagotomy (Fig. 5b). Vagotomy resulted in significant reduction in food intake (*P* < 0.0001) (Fig. 5c).

### Effect of reduced food intake on insulin secretion (experiment 6)

To estimate the effect of reduced food intake of vagotomized rats on amino acid-induced insulin secretion, we fed rats with 2.5 g of the 20P diet for 1 h as a restricted feeding group (RF), which is almost the same amount of food consumed in the vagotomized groups in Experiment 5. Rats in the ad libitum group (AL) were fed 20P diet freely for 1 h (Fig. 5f). Plasma insulin and glucose concentrations were not affected by the reduced food intake (Fig. 5d, e).

## Discussion

In the present study, we investigated which amino acid was responsible for the reduced insulinotropic activity of the low-protein diet. Results of plasma amino acid concentration during an absorptive state suggest that feeding low-protein diet reduced plasma essential amino acids and indicated that molecular signals of eating protein-deficient diet are immediately recognized by the digestive tract and reflected in plasma amino acid levels. Therefore, we further investigated which amino acid was responsible for the reduced insulinotropic activity of the low-protein diet. Results of single amino acid deprivation demonstrated that feeding for at least 2 h with diets deprived of Val, Leu, or Ile prominently reduced insulin secretion while deprivation of other amino acids was ineffective. This result suggested that a reduced BCAA content could be the cause for decreased insulinotropic activity of the low-protein diet; however, reduced food consumption might contribute to the reduction in insulin secretion in the case of Leu or Ile deprivation. The reduced food intake in BCAA-deficient animals was consistent with a previous report [30]. Then, diets with reduced BCAA to 25% of the control diet, which did not induce reduction of food intake, were used in the next experiment. Based on the results that insulin secretion was still low in Leu-restricted group, we concluded that Leu and Val are the only amino acids whose deficiency reduces insulin secretion to the level seen in the low-protein diet-fed animals in vivo. Since insulin concentration in pancreatic islets was not decreased in rats fed a low-protein diet for 1 day (data not shown), amino acid-deficient diet possibly impairs insulin secretion without reducing insulin content in beta cells. Meanwhile, Leu or Val supplementation did not restore the reduced insulin secretion in the rats fed the low-protein diet; supplementation of three BCAAs at the same time was necessary to fully restore the reduced insulin secretion. The results of these animal experiments suggest that Leu and Val were necessary to maintain normal insulin release and supplementation with the three BCAAs was sufficient to restore insulinotropic activity of the low-protein diet.

Although insulin secretion was significantly decreased by feeding amino acid-deficient diets, plasma glucose concentrations did not change. One possible explanation is that plasma insulin concentration induced by an amino acid-deficient diet is low but sufficient to reduce plasma glucose because glucose-induced insulin secretion was not impaired. In other words, amino acids in diet upregulate glucose-stimulated insulin secretion more than necessary to reduce plasma glucose levels. Another possibility is that an amino acid-deficient diet enhances insulin sensitivity and a normal plasma glucose level is maintained regardless of the low plasma insulin levels. Upregulation of insulin sensitivity in rats fed a low-protein diet for 1 week has been previously reported [4, 5]; however, whether upregulation of insulin sensitivity has an immediate effect on plasma glucose levels during eating is unknown.

Because plasma concentrations 30 min after ingestion of BCAAs correlated well with their contents in the diets and plasma insulin level, we hypothesized that BCAAs in plasma synergistically stimulate insulin release from beta cells. We demonstrated that essential amino acids enhanced glucose-induced insulin secretion, and at higher concentrations, essential amino acids stimulated insulin secretion in a glucose-independent manner in MIN6 cells. These results are mostly consistent with the report that each BCAA directly stimulated insulin secretion and that Leu was the most effective among the three BCAAs, as reported previously in INS-1E, BRIN-BD11, and MIN6 cells [31–33]. However, a synergistic insulinotropic effect of the three BCAAs was not observed in MIN6 cells. Therefore, we conclude that plasma BCAA directly upregulates insulin secretion from beta cells, and three BCAAs synergistically stimulate insulin secretion through a distinct mechanism.

As a possible mechanism for the synergistic insulinotropic effect of the three BCAAs, we examined involvement of the vagus nerve system because insulin secretion is regulated by afferent and efferent vagus nerve activity [17, 18]. Furthermore, amino acids activated or inactivated vagal hepatic afferents when administered intraportally [34, 35], and supplementation of Leu to a low-protein diet activated the vagus nerve and appetite [29]. These results indicate the possibility that reduced BCAA intake exerts immediate effects on vagus nerve activity and subsequent insulin secretion. The present result that enhancement of insulin secretion in 20P and 5P + BCAA groups disappeared in vagotomized rats demonstrates the possibility that synergistic insulinotropic effect of BCAAs is exerted through vagus nerve activity. Moreover, the result that vagotomy did not reduce insulin secretion of rats fed a low-protein diet demonstrated that the vagus nerve was not a main regulator of glucose-induced insulin secretion.

In vagotomized rats, food consumption was significantly reduced, which possibly blunted the effect of amino acids to stimulate insulin secretion. Reduction of food consumption in vagotomized rats was reported and attributed to loss of satiety regulation and suppression of the hunger-promoting signals [36, 37]. To clarify the possibility that reduced food consumption impaired insulinotropic activity of amino acids, we restricted food intake of the rats to the level of vagotomized groups and demonstrated that the reduced food intake did not impair amino acid-induced insulin secretion during the absorptive state. From these results, we finally conclude that insulinotropic effect of three BCAAs was exerted via a vagus nerve-dependent mechanism. Although the precise mechanism is unknown at present, one possible mechanism is that BCAAs in ingested foods directly activate the vagal afferent nerve in the intestine and that the efferent vagus nerve simulates insulin secretion via the M3 muscarinic receptor on the surface of beta cells [17, 18]. It is also possible that BCAAs activate GLP-1 secretion from intestine, which was reported to increase insulin secretion via activation of the vagus nerve system or directly via a specific cell surface receptor [17, 38].

Vagotomy also induced hypersecretion of insulin in response to feeding in some rats. Hyperinsulinemia of vagotomized rats fed a liquid meal was reported and attributed to the reduced gastric retention of liquid meals [39]. Although the reason for insulin hypersecretion in this experiment is unknown, functions of the gastrointestinal tract might be influenced by the vagotomy.

## Conclusions

In the animal experiments of this study, we demonstrated that Leu and Val are the only amino acids whose deficiency reduced insulin secretion and supplementation of three BCAAs was sufficient to restore the reduced insulin secretion of the low-protein diet. From these results, we conclude that decreased insulinotropic activity induced by the low-protein diet is attributed to a reduced BCAA content. The present finding suggests that the three BCAAs have a strong synergistic effect that stimulates insulin secretion via the vagus nerve system in addition to the direct stimulatory effect of each BCAA on beta cells.

## Abbreviations

Ala: Alanine
ANOVA: Analysis of variance
Arg: Arginine
BCAAs: Branched-chain amino acids
CCK: Cholecystokinin
DMEM: Dulbecco’s Modified Eagle’s medium
ELISA: Enzyme-linked immunosorbent assay
Gln: Glutamine
Glu: Glutamic acid
Ile: Isoleucine
Leu: Leucine
Lys: Lysine
mTOR: Mammalian target of rapamycin
Phe: Phenylalanine
Pro: Proline
TCA: Tricarboxylic acid
Val: Valine
